# Metformin inhibits mitochondrial adaptations to aerobic exercise training in older adults

**DOI:** 10.1111/acel.12880

**Published:** 2018-12-11

**Authors:** Adam R. Konopka, Jaime L. Laurin, Hayden M. Schoenberg, Justin J. Reid, William M. Castor, Christopher A. Wolff, Robert V. Musci, Oscar D. Safairad, Melissa A. Linden, Laurie M. Biela, Susan M. Bailey, Karyn L. Hamilton, Benjamin F. Miller

**Affiliations:** ^1^ Department of Kinesiology and Community Health University of Illinois Urbana‐Champaign Urbana Illinois; ^2^ Department of Health and Exercise Science Colorado State University Fort Collins Colorado; ^3^ Department of Environmental & Radiological Health Sciences Colorado State University Fort Collins Colorado; ^4^ Aging and Metabolism Research Program Oklahoma Medical Research Foundation Oklahoma City Oklahoma

**Keywords:** aging, healthspan, protein synthesis, proteostasis, telomere

## Abstract

Metformin and exercise independently improve insulin sensitivity and decrease the risk of diabetes. Metformin was also recently proposed as a potential therapy to slow aging. However, recent evidence indicates that adding metformin to exercise antagonizes the exercise‐induced improvement in insulin sensitivity and cardiorespiratory fitness. The purpose of this study was to test the hypothesis that metformin diminishes the improvement in insulin sensitivity and cardiorespiratory fitness after aerobic exercise training (AET) by inhibiting skeletal muscle mitochondrial respiration and protein synthesis in older adults (62 ± 1 years). In a double‐blinded fashion, participants were randomized to placebo (*n* = 26) or metformin (*n* = 27) treatment during 12 weeks of AET. Independent of treatment, AET decreased fat mass, HbA1c, fasting plasma insulin, 24‐hr ambulant mean glucose, and glycemic variability. However, metformin attenuated the increase in whole‐body insulin sensitivity and VO_2_max after AET. In the metformin group, there was no overall change in whole‐body insulin sensitivity after AET due to positive and negative responders. Metformin also abrogated the exercise‐mediated increase in skeletal muscle mitochondrial respiration. The change in whole‐body insulin sensitivity was correlated to the change in mitochondrial respiration. Mitochondrial protein synthesis rates assessed during AET were not different between treatments. The influence of metformin on AET‐induced improvements in physiological function was highly variable and associated with the effect of metformin on the mitochondria. These data suggest that prior to prescribing metformin to slow aging, additional studies are needed to understand the mechanisms that elicit positive and negative responses to metformin with and without exercise.

## 1 INTRODUCTION

The declines in cardiorespiratory fitness (CRF), glucose control, and insulin sensitivity are predictors of disease, disability, and all‐cause mortality (Blair et al., 1989; Facchini, Hua, Abbasi, & Reaven, 2001; Zaslavsky, Walker, Crane, Gray, & Larson, 2016). The age‐related loss of CRF and insulin sensitivity is associated with lower mitochondrial protein synthesis (biogenesis), abundance, and respiration and elevated reactive oxygen species emissions (ROS) (Anderson et al., 2009; Rooyackers, Adey, Ades, & Nair, 1996; Short et al., 2005). Additionally, the decline in mitochondrial function is directly linked with telomere shortening (Sahin et al., 2011). Telomeres, protective caps at the ends of chromosomes, are critical features for maintaining genome integrity and stability in response to a variety of cellular stresses (e.g., cell division and oxidative stress) and lifestyle factors (e.g., nutrition and exercise). Therefore, telomeres may provide an integrated cellular measure of general health and biological aging.

Aerobic exercise is considered the gold standard approach to increase CRF and peripheral insulin sensitivity across the lifespan. Findings from prospective studies suggest a greater CRF and insulin sensitivity impart protection from the onset of age‐related chronic disease (Blair et al., 1989; Facchini et al., 2001). While the molecular transducers that give rise to the pleiotropic health benefits of exercise are still being elucidated, the energetic stress of exercise increases AMP‐activated kinase (AMPK) activity (See Richter & Ruderman, 2009 for review). AMPK is considered an energy sensor of the cell and, in response to energetic stress, increases energy‐producing processes such as skeletal muscle mitochondrial biogenesis and insulin‐stimulated glucose uptake. In line with this notion, we and others have shown that increased skeletal muscle mitochondrial protein synthesis and respiration as well as decreased ROS emissions may contribute to improved CRF and peripheral insulin sensitivity after aerobic exercise training (AET; Konopka et al., 2015; Robinson et al., 2017).

The biguanide metformin is the most widely prescribed medication to treat type 2 diabetes mellitus (T2DM), and there is also a growing interest in using metformin to treat aging and delay the onset of multiple age‐related diseases (Barzilai, Crandall, Kritchevsky, & Espeland, 2016). Metformin decreases hepatic glucose production (Hundal et al., 2000) and increases peripheral glucose disposal in adults with and without T2DM (Malin, Gerber, Chipkin, & Braun, 2012; Musi et al., 2002). Collectively, the glucose‐lowering effect of metformin appears, like exercise, to be through an energetic stress and/or redox mechanism in both skeletal muscle and liver. Metformin inhibits mitochondrial respiration at complex I (CI) of the electron transport system (Brunmair et al., 2004; Wessels, Ciapaite, van den Broek, Nicolay, & Prompers, 2014), which stimulates an energetic stress in skeletal muscle and activates AMPK for glucose uptake (Malin et al., 2012; Musi et al., 2002). Additionally, metformin may improve insulin sensitivity by decreasing skeletal muscle mitochondrial ROS emissions (Kane et al., 2010). Therefore, within skeletal muscle, the mechanisms of action for metformin appear to be due to an inhibitory effect at the level of the mitochondria which alter the energetic and/or redox status of the cell.

The U.S. Diabetes Prevention Program (USDPP) demonstrated that both lifestyle modification, including 150 min of moderate intensity exercise per week, and metformin independently prevented the progression from prediabetes to T2DM by 58% and 31%, respectively (Knowler et al., 2002). Several studies have since tested the effects of metformin plus exercise to determine whether the combination would elicit favorable synergistic or unfavorable antagonistic effects on physiological function implicated in age‐related chronic disease. Evidence now suggests that the addition of metformin to acute or chronic exercise does not induce an additive effect but instead inhibits the exercise‐mediated improvements in CRF and insulin sensitivity (Malin et al., 2012; Sharoff et al., 2010). The mechanisms mediating the antagonistic interaction between metformin and exercise on physiological function involved in healthy aging are unknown.

Aerobic exercise training and metformin independently have positive effects on the improvement of whole‐body and peripheral insulin sensitivity (Konopka et al., 2015, 2016; Malin et al., 2012; Musi et al., 2002; Robinson et al., 2017) through overlapping yet distinct cellular mechanisms. A classic adaptation to the energetic demands of AET is to increase the capacity for energy production through mitochondrial biogenesis and mitochondrial respiration while metformin generates an energetic stress by inhibiting mitochondrial respiration with unknown effects on mitochondrial biogenesis. Due to the juxtaposition of metformin and AET at the level of the mitochondria, we tested the hypothesis that metformin restricts the increase in CRF and insulin sensitivity after 12 weeks of AET in older adults free of chronic disease by inhibiting the increase in skeletal muscle mitochondrial respiration and protein synthesis.

## 2 RESULTS

### 2.1 Physical and clinical characteristics

All participants were free of chronic disease but had at least one risk factor for T2DM: fasting glucose 100–125 mg/dl, 2‐hr glucose 140–199 mg/dl, HbA1c (5.7%–6.4%), or family history of T2DM. Forty‐two participants (80%) had family history of T2DM, and 14 (26%) were taking statins throughout the study. All participants were metformin naïve. The dose was decreased from 2,000 to 1,500 mg/day in 11 participants who reported gastrointestinal discomfort (placebo *n* = 3; metformin *n* = 8). Pill compliance was 98% as calculated by returned pill count. Table 1 shows the participants’ physical and clinical characteristics. The 12 weeks of AET decreased (*p* < 0.05) body weight, independent of metformin or placebo, which was largely due to the reduction (*p* < 0.001) in whole body, trunk, and leg fat mass. Fat‐free mass was not statistically different after the intervention.

**Table 1:** Participant physical and clinical characteristics

	Placebo		Metformin		Main effect for time (*p*)
	PRE	POST	PRE	POST	
*N*	26 (21 W, 5 M)		27 (21 W, 6 M)		
Age	63 ± 1		62 ± 1		
Body weight (kg)	84 ± 4	83 ± 3	86 ± 4	84 ± 4	<0.001
BMI	30 ± 1	30 ± 1	31 ± 1	30 ± 1	–
Fat mass (kg)	34 ± 2	33 ± 2	34 ± 2	32 ± 2	<0.001
Trunk fat mass (kg)	18 ± 2	16 ± 1	18 ± 1	16 ± 1	<0.001
Leg fat mass (kg)	12 ± 1	11 ± 1	11 ± 1	10 ± 1	<0.001
Fat‐free mass (kg)	47 ± 2	47 ± 2	49 ± 2	48 ± 2	–
HbA1c (%)	5.8 ± 0.06	5.7 ± 0.05	5.7 ± 0.07	5.6 ± 0.06	<0.01
Fasting glucose (mg/dl)	96 ± 1	95 ± 2	104 ± 3	103 ± 2	–
Fasting insulin (µIU/ml)	8.3 ± 1.0	6.1 ± 0.7	10.1 ± 2.1	8.3 ± 1.8	<0.05
HOMA‐IR	1.8 ± 0.3	1.2 ± 0.2	2.4 ± 1.1	1.8 ± 0.7	<0.05
Glucose AUC	17,115 ± 486	16,734 ± 554	18,821 ± 692	19,647 ± 530	–
Insulin AUC	8,707 ± 1,355	7,012 ± 1,033	9,617 ± 1,420	9,988 ± 2,142	–
2‐hr glucose (mg/dl)	109 ± 7	107 ± 10	118 ± 7	133 ± 6	–
2‐hr insulin (µIU/ml)	56 ± 9	53 ± 8	60 ± 10	62 ± 17	–

Note

AUC, area under the curve; BMI, body mass index; HOMA‐IR, homeostatic model assessment of insulin resistance.

When no *p* values are provided, *p* > 0.05. *p* < 0.05 depicts the main effects of time. No effect of treatment or interaction was present.

### 2.2 Cardiorespiratory fitness, whole‐body insulin sensitivity, and glucose regulation

**Figure 1:**
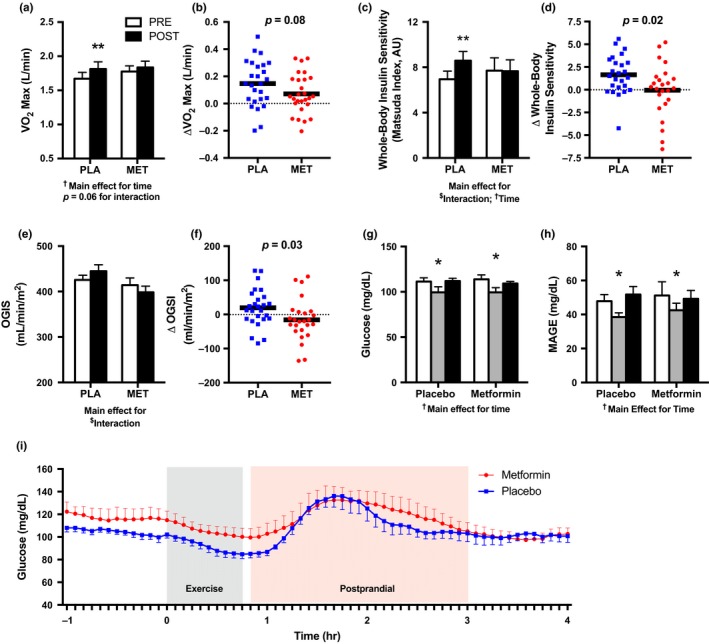
Metformin attenuates cardiorespiratory fitness and whole‐body insulin sensitivity after 12 weeks of AET. (a) VO_2_ max (L/min) before and after 12 weeks of AET with placebo (PLA) or metformin (MET). PLA (*n* = 26), MET (*n* = 27). (b) The change (Δ) in VO_2_ max (L/min) after 12 weeks of AET with PLA vs. MET. (c) Whole‐body insulin sensitivity before and after 12 weeks of AET with PLA or MET. PLA (*n* = 24), MET (*n* = 25). (d) The change (Δ) in whole‐body insulin sensitivity after 12 weeks of AET with PLA vs. MET. (e) Oral glucose insulin sensitivity (OGIS) index before and after 12 weeks of AET with PLA vs. MET. (f) The change (Δ) in OGIS after 12 weeks of AET with PLA vs. MET. Continuous glucose monitor (CGM) derived (g) 24‐hr mean glucose and (h) mean amplitude of glucose excursions (MAGE) before, at week 6, and after 12 weeks of AET with placebo or metformin. (i) A 5‐hr duration to capture glucose behavior during standardized exercise and post‐exercise nutrition. Post‐exercise nutrition was consumed immediately after the exercise. CGM was used in a subset of participants; PLA (*n* = 9), MET (*n* = 8). *p < 0.05 vs. PRE, ** *p* < 0.01 vs. PRE. AET: aerobic exercise training; MET: metformin; PLA: placebo. Data are presented as mean ± *SEM*

Figure 1a demonstrates that VO_2_max (L/min) was significantly increased after 12 weeks of AET (*p* < 0.05, main effect for time) with a significant increase within the placebo group (*p* < 0.01) but not metformin. Metformin attenuated the increase in VO_2_max following 12 weeks of AET by ~50%, although this did not reach statistical significance (*p* = 0.08, Figure 1b). After an overnight fast, subjects completed a blood chemistry assessment and oral glucose tolerance test (OGTT). HbA1c (%), fasting insulin, and HOMA‐IR were decreased (*p* < 0.05) after 12 weeks of AET, independent of metformin or placebo (Table 1). There was a significant interaction (*p* < 0.05, time by treatment) for an increase in whole‐body insulin sensitivity, evaluated by the Matsuda index, after AET with placebo but not metformin (Figure 1c). On average, the increase in whole‐body insulin sensitivity after AET was inhibited (*p* = 0.02) by metformin (Figure 1d). The change in insulin sensitivity after AET with metformin was highly variable compared to placebo as evident by approximately twice the *SE* (4.0 vs. 2.1). Further, there was a dichotomous response to AET with metformin where 58% of participants were positive responders with increased insulin sensitivity and 42% responded negatively with decreased insulin sensitivity (Figure 1d). Similarly, the oral glucose sensitivity index (OGSI) (Figure 1e,f), an estimate of glucose clearance, followed a similar pattern as the Matsuda index where there was a significant interaction (*p* < 0.05, time by treatment) between placebo and metformin. Postprandial glucose and insulin area under the curve (AUC) were not different after the intervention, independent of placebo or metformin.

To further assess glucose regulation, we used continuous glucose monitors (CGM) in a subset of 17 individuals (placebo *n* = 9; metformin *n* = 8) for ~7 days on three separate occasions, before, during, and after the intervention. Interstitial glucose was measured every 5 min (288 measurements per 24 hr) to determine several indices of glucose behavior and variability. Compared to before AET, we found that 24‐hr mean ambulant glucose and mean amplitude of glycemic excursions (MAGE), an index of postprandial glucose excursions, were lower during (*p* < 0.05), but no longer statistically different after AET, independent of placebo or metformin (Figure 1g,h). Since these measurements are taken during free‐living situations where diet was not standardized, we also performed an exploratory analysis of glucose behavior for the 5 hr that consisted of the standardized aerobic exercise session and post‐exercise nutrition as shown in Figure 1i. We observed the expected exercise‐induced decline and postprandial rise in glucose, but we did not detect a statistical difference in glucose AUC between groups.

### 2.3 Skeletal muscle mitochondrial respiration

**Figure 2:**
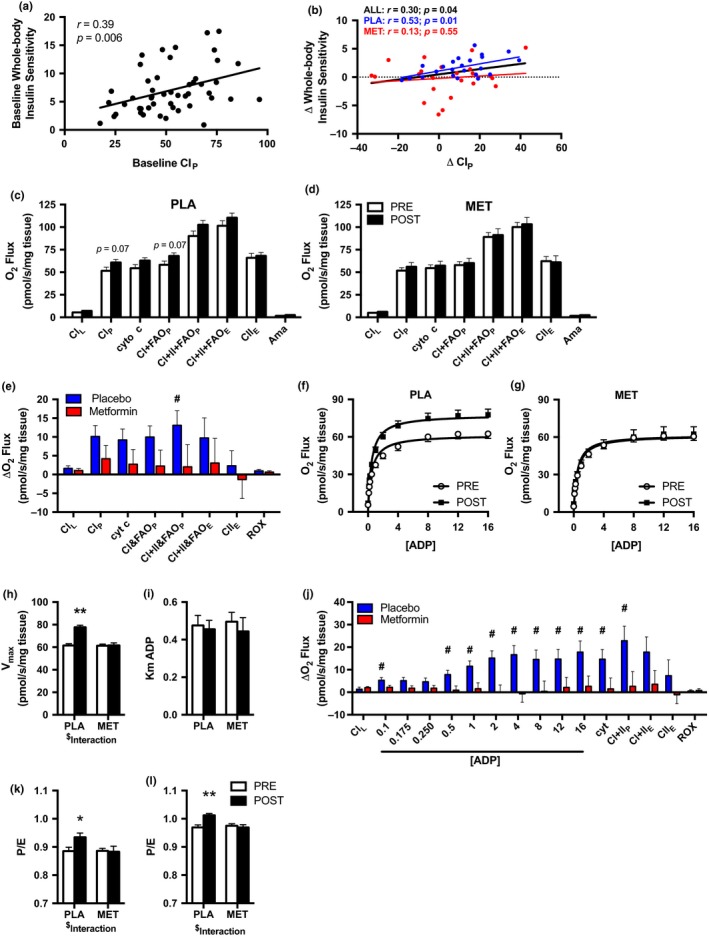
Metformin prevents the increase in skeletal muscle mitochondrial respiration after 12 weeks of AET. Association of whole‐body insulin sensitivity and CI_P_ at (a) baseline and (b) with the change (Δ) from pre‐ to postintervention. Mitochondrial respiration before and after 12 weeks of AET with (c) placebo (PLA) and (d) metformin (MET) during the SUIT1 protocol. The change (Δ) in mitochondrial respiration for (e) SUIT1. ADP titration curve before and after 12 weeks of AET with (f) PLA or (g) MET. Using Michaelis–Menten Kinetics, (h) *V*_max _and (i) the apparent Km of ADP were calculated before and after 12 weeks of AET with PLA or MET. The change (Δ) in mitochondrial respiration for (j) the ADP titration protocol. The ratio of maximal coupled OXPHOS to uncoupled ETS (P/E) for the (k) SUIT1 and (l) ADP titration protocol before and after 12 weeks of AET with PLA or MET. ^#^ *p* < 0.05 vs. metformin; *p < 0.05, **p < 0.01 vs. PRE. PLA (*n* = 24), MET (*n* = 26). Data are presented as mean ± *SEM*

Skeletal muscle biopsies were obtained from the vastus lateralis to evaluate mitochondrial respiration in permeabilized muscle fibers. To determine the chronic adaptation to the intervention while avoiding the acute influence of exercise and metformin, muscle biopsies were obtained 48 hr after the last exercise bout and 36 hr after the last metformin dose. Since a putative role of metformin is to inhibit complex I (CI), we used two protocols to assess mitochondrial respiration that was initiated by providing CI‐linked substrates pyruvate, glutamate, and malate. In the first protocol (SUIT1), we used an ADP bolus to stimulate maximal oxidative phosphorylation (OXPHOS; P) to evaluate CI_P_ followed by provision of octanoylcarnitine and succinate to assess fatty acid oxidation (CI&FAO_P_) and complex II‐linked (CI+II&FAO_P_) respiration, respectively. Last, the protonophore FCCP was added to stimulate maximal uncoupled electron transport system capacity (ETS; CI+II&FAO_E_). Before the intervention, CI_P_ was correlated with whole‐body insulin sensitivity (Figure 2a). After 12 weeks of AET, the change in CI_P_ was also correlated with the change in whole‐body insulin sensitivity (Figure 2b). The relationship between the change in CI_P_ and whole‐body insulin sensitivity was primarily driven by the differences observed after AET when taking placebo rather than metformin. With placebo, AET nonsignificantly increased CI_P_ and CI+FAOP (both *p* = 0.07) and this trend was not apparent with metformin (Figure 2c,d). Further, the change in CI+II&FAO_P_ was greater (*p* < 0.05) in placebo versus metformin (Figure 2e).

In the second protocol (ADP titration), we titrated ADP to stimulate submaximal and maximal CI_P_ followed by CI+II_P_ and CI+II_E_. From the ADP titration curve, we estimated the apparent ADP *K*_m_ and *V*_max_ using Michaelis–Menten kinetics. The AET‐induced increases in submaximal and maximal CI‐linked respiration (Figure 2f,g) as well as *V*_max_ (Figure 2h) were abolished by metformin. In contrast, the apparent ADP *K*_m_ was not different between placebo and metformin after the 12‐week intervention (Figure 2i). The change in mitochondrial submaximal and maximal CI‐linked and CI+II_P_ respiration after 12 weeks of AET was significantly different with placebo vs. metformin (Figure 2j).

The ratio of maximal coupled OXPHOS to uncoupled ETS capacity (P/E) represents an index of intrinsic mitochondrial function and describes OXPHOS as a limiting factor in ETS capacity. In both SUIT1 and ADP titration protocols, there was an interaction (*p* < 0.05; time by treatment) suggesting metformin blunted the AET‐induced increase in P/E observed in the placebo group (Figure 2k,l).

### 2.4 Skeletal muscle telomere length

**Figure 3:**
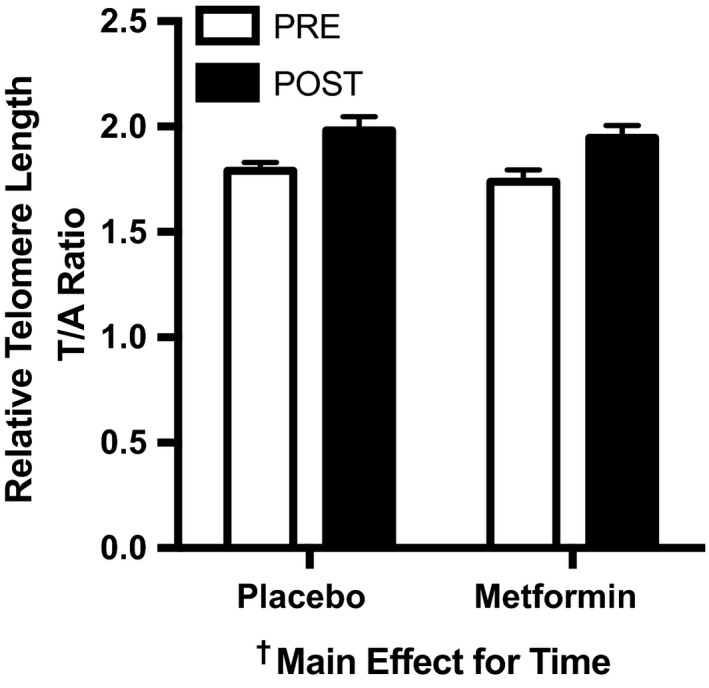
Skeletal muscle telomere length was increased after 12 weeks of AET. Skeletal muscle telomere length before and after 12 weeks of AET with PLA or MET. Data are presented as mean ± *SEM*. PLA (*n* = 22); MET (*n* = 25)

Since telomeres regulate genome stability and are directly linked with mitochondrial function, we aimed to understand whether exercise with or without metformin may influence telomere length. Skeletal muscle telomere length was measured in 47 study participants (22 placebo; 25 metformin) using qRT–PCR. Telomere length was significantly increased after 12‐weeks of AET (*p* < 0.001) independent of metformin or placebo treatment (Figure 3).

### 2.5 Skeletal muscle protein synthesis and nutrient sensing

**Figure 4:**
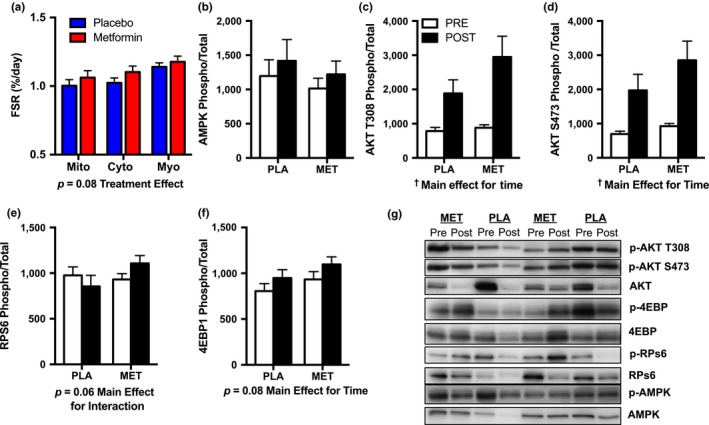
Skeletal muscle protein synthesis rates and nutrient‐sensing signaling proteins. (a) Cumulative muscle protein synthesis rates in subcellular fractions enriched for mitochondrial (Mito), cytoplasmic (cyto), and myofibrillar (myo) proteins during the last 4‐week AET with placebo versus metformin. PLA (*n* = 24), MET (*n* = 26) (b) AMPK, (c) AKT T308, (d) AKT S473, (e) RPS6, and (f) 4EBP1 before and after AET with PLA or MET. Proteins were analyzed in a subset of participants due to limited tissue availability, PLA: *n* = 15; Met *n* = 18; (g) representative western blots of each protein of interest. FSR: fractional synthesis rates. All proteins of interest are expressed as phosphorylation relative to total (phosphor/total). Data are presented as mean ± *SEM*

Cumulative skeletal muscle protein synthesis rates were measured during the last 4 weeks of the intervention in subcellular fractions enriched with mitochondrial, cytoplasmic, and myofibrillar proteins. There were no significant differences between placebo and metformin in synthesis rates for any subcellular muscle protein fraction (Figure 4a).

We probed for several nutrient‐sensing proteins involved in adjusting skeletal muscle protein synthesis and insulin action. In the current study, there were no significant differences in AMPK phosphorylation/total (Figure 4b). We also assessed the activation of AKT at two binding sites, T308 and S473, since they are upstream signaling proteins involved in protein synthesis and insulin action. AET increased (*p* < 0.05, main effect for time) AKT phosphorylation/total at T308 and S473 independent of placebo or metformin (Figure 4c,d). 4eBP1 and RpS6 are downstream of AKT phosphorylation/total and mTOR and involved in protein translation initiation and regulation. We observed a trend (*p* = 0.08, main effect of time) for 12 weeks of AET to increase 4eBP1 phosphorylation/total and a trend for an interaction (*p* = 0.06, time by treatment) for RpS6 phosphorylation/total Figure 4e,f. Representative western blots are shown in Figure 4g.

## 3 DISCUSSION

This study shows in older adults at risk for T2DM, but free of chronic disease that a clinical dose of metformin inhibited the improvement in skeletal muscle mitochondrial respiration and attenuated the increase in CRF and whole‐body insulin sensitivity after AET. The change in insulin sensitivity was correlated with the change in CI‐linked respiration after AET in the placebo but not metformin group. Collectively, these data suggest that metformin attenuated the improvements in whole‐body physiological function after 12 weeks of AET, in part, by preventing the increase in skeletal muscle mitochondrial respiration without affecting mitochondrial protein synthesis. Metformin did not diminish the improvements in HbA1c, fasting insulin, 24‐hr mean glucose, and fat mass or the increase in skeletal muscle telomere length after AET. Since CRF and insulin sensitivity are primary predictors of age‐related morbidity and mortality, these data raise concerns about broad recommendations for the use of metformin as a treatment to target aging.

### 3.1 Metformin attenuates the improvement in physiological function after aerobic exercise training

Cardiorespiratory fitness is one of the strongest risk factors for disease and mortality and high levels of CRF impart protection against development of cardiometabolic disease (Blair et al., 1989). CRF decreases with age while AET is commonly recommended to increase CRF and delay the onset of cardiometabolic disease during advancing age. Our findings in older adults at risk for T2DM demonstrate that compared to placebo, metformin blunted the improvement in CRF by ~50% after 12 weeks of AET. These data confirm previous reports that metformin also tended to diminish the increase in CRF by ~40%–60% in middle‐aged prediabetic and T2DM after 10 and 22 weeks of exercise training, respectively (Boulé et al., 2013; Malin et al., 2012). Within these previous studies, the inhibitory effect of metformin was explicitly observed with exercise training while there was no influence of metformin on CRF in sedentary control subjects. Additionally, metformin decreased CRF in healthy, active adults (Braun et al., 2008). Collectively, these results indicate a consistent pattern whereby metformin appears to blunt the increase in CRF after exercise training.

In the current study, we show that metformin inhibits the aerobic exercise‐induced increase in whole‐body insulin sensitivity estimated during a 75 g oral glucose tolerance test. Postprandial insulin sensitivity incorporates both hepatic and peripheral tissues, but the majority is determined by peripheral glucose disposal (DeFronzo et al., 1981). Although exercise and metformin independently improve insulin sensitivity (Konopka et al., 2015, 2016; Malin et al., 2012; Musi et al., 2002; Robinson et al., 2017), our findings are consistent with previous reports that metformin attenuated the increase in peripheral insulin sensitivity by 30%–50% when assessed by a hyperinsulinemic–euglycemic clamp after both an acute exercise bout and 12 weeks of exercise training (Malin et al., 2012; Sharoff et al., 2010). While the overall change in whole‐body insulin sensitivity after AET with metformin was not different from baseline, we did observe a divergent response where nearly half of the participants increased and half of the participants decreased insulin sensitivity.

Emerging evidence suggests that acute exercise plus metformin therapy may result in a lower postprandial glucose excursion compared to exercise or metformin alone (Erickson, Little, Gay, McCully, & Jenkins, 2017). In the current study, besides consuming standardized nutrition immediately after exercise, we did not control for timing of dietary intake or metformin consumption in relation to their exercise bout. It is therefore plausible that the timing of metformin, nutrition, and exercise needs to be highly controlled to minimize the inhibitory effects of metformin on exercise‐induced improvements of insulin sensitivity. However, our findings in participants who wore CGM are not in line with this notion as we demonstrated a decrease in 24‐hr mean glucose and glycemic variability during the intervention, independent of metformin or placebo treatment. The decrease in 24‐hr glucose during AET was lost during the 7 days after the last exercise training bout which is consistent with previous work that demonstrates the glucoregulatory benefits of exercise dissipate after 3 days of inactivity (King et al., 1995). Elevated glucose in nondiabetic individual (110–115 mg/dl) was associated with increased risk of age‐related conditions such as dementia and frailty compared to those with normal glucose values (<100 mg/dl; Crane et al., 2013; Zaslavsky et al., 2016). Conversely, individuals with a propensity for long life had a lower 24‐hr mean glucose compared to normal age‐matched controls (Wijsman et al., 2013). Additional research is needed to determine whether lowering mean glucose, glycemic variability, or both are critical factors involved in mediating healthy longevity in humans. Even though we find that metformin diminished two key positive health benefits of exercise, CRF and insulin sensitivity, metformin did not hinder nor accentuate the improvement in 24‐hr mean glucose, HbA1c, or fasting plasma insulin with AET. These data suggest that the effects of metformin are highly variable not only within a given outcome like insulin sensitivity but also across outcomes related to glucoregulation.

### 3.2 Metformin abolishes the improvement in mitochondrial respiration after aerobic exercise training

We report that the correlation between the change in CI‐linked mitochondrial respiration and insulin sensitivity after AET was driven by the relationship in the placebo (*r* = 0.53) group rather than metformin (*r* = 0.13), which suggests that metformin may act on the mitochondria at CI to disrupt the relationship between insulin sensitivity and mitochondrial respiration. Our findings largely indicate that metformin inhibits the increase in submaximal and maximal CI‐linked skeletal muscle mitochondrial respiration after AET. Metformin abolished the ~25% increase in maximal CI‐linked respiration after AET during the ADP titration. No other studies have evaluated the impact of metformin on mitochondrial adaptations to aerobic exercise, but our findings are consistent with the 21% decrease in maximal CI‐linked respiration in isolated mitochondria from lean and diabetic sedentary rats treated with metformin (100 mg kg^−1^ day^−1^) (Wessels et al., 2014). Although not all in vivo studies demonstrate a decrease in maximal CI‐linked respiration by metformin (Kane et al., 2010), numerous in vitro studies (Brunmair et al., 2004; Owen, Doran, & Halestrap, 2000; Wheaton et al., 2014) support the premise that metformin inhibits CI of the ETS. Metformin decreases cancer cell and fibroblast proliferation by inhibiting CI of the ETS (Wheaton et al., 2014). By overexpressing NDI1, a subunit within CI that oxidizes NADH, cells were no longer susceptible to the inhibitory effect of metformin (Wheaton et al., 2014). Collectively, these data support the concept that one mechanism of action for metformin is linked to CI of the mitochondria.

The increase in maximal and submaximal mitochondrial respiratory capacity after AET can result from improved intrinsic mitochondrial function and/or increased abundance of mitochondria. By adding FCCP to uncouple OXPHOS and ATP synthesis, we can determine the contribution of OXPHOS to maximal ETS capacity as an index of intrinsic mitochondrial function to infer whether changes in respiration were independent from mitochondrial abundance. The prevailing hypothesis is that a low P/E represents a reserve capacity that is not being utilized and may be an indicator of poor coupling efficiency. We have previously shown that a low P/E is observed in the sedentary or untrained state and increases with exercise (Konopka et al. 2017; Miller et al., 2017). In the current study, the improvement in P/E after AET was inhibited by metformin which suggests that metformin acts directly on the ETS to alter intrinsic mitochondrial function independently of influencing mitochondrial biogenesis or abundance.

In addition to respiration, the mitochondria also produce reactive oxygen species (ROS). Since both exercise (Konopka et al., 2015) and metformin (Kane et al., 2010; Madiraju et al., 2014) have been shown to alter mitochondrial respiration, ROS emissions, and/or cellular redox status, we sought to evaluate an integrated outcome influenced by all these variables. Telomere length, a well‐established indicator of cellular aging, has been directly linked to compromised mitochondrial function and oxidative stress (Sahin et al., 2011; von Zglinicki, 2000). Previous cross‐sectional studies have demonstrated greater telomere length in leukocytes and skeletal muscle of endurance exercise‐trained adults (LaRocca, Seals, & Pierce, 2010; Østhus et al., 2012), we show for the first time that just 12 weeks of AET increased skeletal muscle telomere length, independent of metformin treatment. These data add to the evidence that exercise is an effective geroprotective intervention.

### 3.3 Influence of metformin on skeletal muscle protein synthesis and nutrient sensing

Aerobic exercise and metformin have been shown to alter key intracellular nutrient‐sensing pathways. We reasoned that the cellular energy sensor AMPK would have increased activity due to the energetic stress of metformin preventing the increase in mitochondrial respiration in response to the energetic demands of AET. The influence of exercise and metformin on skeletal muscle AMPK activation is transient, and effects on this signaling node were likely lost by 48 hr after exercise and 36 hr after the last metformin dose (Richter & Ruderman, 2009). In contrast to AMPK, AET increased AKT T308 and S473 which suggests the blunted improvement in whole‐body insulin sensitivity after AET is not linked to an impaired increase in skeletal muscle insulin signaling at AKT.

We also evaluated skeletal muscle protein translation and associated regulatory proteins. The tendency to increase the phosphorylation of 4eBP1 may indicate an increased signal to initiate translation of mRNA into functional proteins 48 hr after the last bout of AET. Intriguingly, we also demonstrated a trend for an interaction for increased phosphorylation of RpS6 after aerobic exercise when taking metformin. RpS6 is controlled via the mTOR pathway and a regulator of ribosome biogenesis. We anticipated that metformin would have blunted the exercise‐induced phosphorylation of RpS6 as previously shown with treatments that activate energetic stress signaling pathways, such as caloric restriction (Miller, Robinson, Bruss, Hellerstein, & Hamilton, 2012) or metformin (Kulkarni et al., 2018). Metformin did not significantly alter cumulative mitochondrial protein synthesis rates that occurred during the last 4 weeks of the 12‐week AET. We chose this period to measure because we reasoned that subjects would have reached a steady‐state response to the exercise and metformin. Because of the difference in respiratory function, these data indicate that either a difference in mitochondrial remodeling occurred in the first 8 weeks that we did not capture, or that metformin has a direct effect on the mitochondria independent of mitochondrial remodeling. The latter is consistent with our findings that suggest metformin alters mitochondrial intrinsic function rather than mitochondrial protein synthesis.

### 3.4 Limitations

While our study sought to address the influence of metformin on exercise‐induced physiological and mitochondrial adaptations, one potential limitation is that we did not include a metformin group alone and relied on previous studies who have already tested the influence of metformin on insulin sensitivity and/or CRF (Konopka et al., 2016; Malin et al., 2012; Sharoff et al., 2010). Further, our study did not include a hyperinsulinemic–euglycemic clamp as the gold standard approach of measuring peripheral insulin sensitivity. However, our data using OGTT‐derived estimates of insulin sensitivity are in line with previous studies who have demonstrated that metformin blunts the improvement in insulin sensitivity after exercise (Malin et al., 2012; Sharoff et al., 2010). Future studies may also include additional groups across a continuum of metabolic health such as individuals who are insulin‐sensitive, insulin‐resistant, and newly diagnosed T2DM.

### 3.5 Considerations for metformin as a treatment to target aging

The results of the current study should be considered in the larger context of proposed treatments to slow aging. Retrospective data and trials in populations with T2DM have shown improved overall survival with metformin (Bannister et al., 2014), as well as decreased risk of cardiovascular disease (UKPDS Group, 1998), cancer incidence (Wu, Boudreau, Park, Simonds, & Freedman, 2014), and cognitive decline (Cheng et al., 2014). Although these studies support the use of metformin as a potential healthspan extending treatment, these findings were largely in subjects with T2DM and none were in adults absent of disease. For metformin to successfully target and delay aging, it should (a) decrease age‐related diseases in general, rather than only the decreased incidence of a single disease (i.e., T2DM), (b) be effective at reducing the onset of age‐related diseases in most, if not all populations, rather than just those with hyperglycemia and T2DM, and (c) prevent or delay the deleterious effects of aging before the accumulation of age‐related morbidities. Our study is important in that it shows metformin can have positive or negative effects on physiological function in those at risk, but absent of chronic disease. The findings lead to questions about whether metformin should be used as an adjunct to an exercise program or in those who are already physically active and have high CRF. In addition, they highlight the need to further explore the effects of metformin in healthy individuals prior to widespread use as a slowed aging treatment.

## 4 CONCLUSION

In summary, our findings show that metformin inhibits the increase in skeletal muscle mitochondrial respiration after 12 weeks of moderate to vigorous AET despite no differences between placebo and metformin on mitochondrial protein synthesis. Metformin also attenuated the increase in whole‐body insulin sensitivity and CRF after AET. However, metformin did not inhibit other AET improvements, including telomere elongation, fasting insulin, 24‐hr mean glucose, and body composition. Our findings suggest that combining two healthspan extending treatments, metformin and exercise, may interfere with the improvement in some parameters of physiological function and do not interact synergistically. This study indicates that further research is needed before broadly prescribing metformin as a treatment to slow aging.

## 5 EXPERIMENTAL PROCEDURES

Details of all study procedures and techniques can be found in the Supporting Information. This study was approved by the Institutional Review Board at Colorado State University (15‐5837H) and registered as a clinical trial (NCT02552355). Prior to beginning the study, participants were informed of the study procedures, risks, and benefits and then provided written consent.

### 5.1 Intervention

All participants completed a supervised, 12‐week AET program with 100% compliance. The AET included three sessions per week and 45 min per session. The first 15 min was at 60% of HR max while the next 30 min was at a specified exercise intensity that progressively increased 5% each week from 65% HRmax to 85% HRmax by week 5. Participants had the option to exercise on a treadmill, stationary upright cycle ergometer, or an elliptical machine. Participants were instructed to not change their normal diet. Immediately after each exercise session, participants consumed a standardized beverage that consisted of carbohydrates (82 g) or carbohydrate (63 g) plus protein (20 g). The original rationale for post‐exercise feeding was to determine whether protein could stimulate skeletal muscle mitochondrial protein synthesis as a way to offset the inhibitory effects of metformin. Since there was minimal influence of post‐exercise nutrition on both clinical and cellular variables, these groups were collapsed to focus on the effects of placebo vs. metformin. Physical and clinical characteristics for those receiving different post‐exercise feeding can be found in Supporting Information Table S1.

In a double‐blinded fashion, participants were randomized to consume either placebo (lactose) or metformin. The dose for both placebo and metformin was 500 mg/day the first week and increased by 500 mg each week until reaching 2000 mg/day (1,000 mg twice daily) by week 4. If participants weighed <75 kg, the maximum dose was 1,500 mg/day (*n* = 5). Participants taking 1,500 mg/day were instructed to consume 500 mg in the morning and 1,000 mg in the evening. Participants were given more pills than needed on a weekly basis during the first 4 weeks and every other week thereafter to monitor pill compliance by the number of pills returned.

### 5.2 Oral glucose tolerance test

All participants arrived to the laboratory after an overnight fast. A venous catheter was inserted to collect serial blood samples before (−10 min) and after (0, 5, 10, 20, 30, 45, 60, 90, and 120 min) consumption of 75 g of glucose. Estimates of whole‐body insulin sensitivity and glucose clearance were calculated using the Matsuda Index (Matsuda & DeFronzo, 1999) and the oral glucose insulin sensitivity (OGIS) index (Mari, Pacini, Murphy, Ludvik, & Nolan, 2001), respectively.

### 5.3 Cardiorespiratory fitness

VO_2_max was determined on a stationary cycle ergometer (Lode), as previously performed (Konopka et al. 2017; Robinson, Turner, Hellerstein, Hamilton, & Miller, 2011). The workload began at 50 Watts and increased every 2 min by 20 Watts for women and 30 Watts for men. Respiratory gases were measured using a ParvoMedics metabolic system.

### 5.4 Skeletal muscle biopsy

A muscle biopsy sample was obtained from the vastus lateralis before and after the 12‐week intervention under local anesthesia (1% lidocaine). Muscle bundles for high‐resolution respirometry were immediately placed in ice‐cold BIOPS buffer while the remaining muscle was frozen in liquid nitrogen and stored at −80C until processed for analysis. Skeletal muscle data are unavailable for one participant because no muscle sample was obtained.

### 5.5 Mitochondrial respiration

Permeabilization and respiration of muscle fibers were performed in duplicate as previously performed (Konopka et al. 2017; Miller et al., 2017). Respiration rates (O_2_ flux) were expressed relative to muscle tissue wet weight (pmol/s/mg tissue).

### 5.6 Telomere length

Multiplexed qPCR measurements of telomere length were carried out as previously described (Cawthon, 2009). Telomere length measurements were performed using a Bio‐Rad CFX‐96 qPCR machine. Multiplexing both telomere and albumin primers using a single fluorescent DNA‐intercalating dye is possible because the telomere sequences are amplified at a lower quantification cycle than the albumin sequences. Standard curves were prepared using human genomic DNA (Promega cat # G3041) with threefold dilutions ranging from 50 to 0.617 ng. Negative controls included a no‐template TelG/C only and AlbU/D only, and a combined TelG/C and AlbU/D control. Samples were normalized across plates using a human genomic DNA standard. Each sample was run in triplicate on a 96‐well plate format, and relative telomere length was established using a telomere (T)‐to‐albumin (A) ratio.

### 5.7 Deuterium labeling

Participants orally consumed deuterium oxide (^2^H_2_O, 70%, Sigma‐Aldrich) to achieve an isotopic steady state of 1%–2% to label newly synthesized skeletal muscle protein. ^2^H_2_O was provided during the last 4 weeks of the intervention (weeks 9–12) starting with a priming stage in week 9 (50 ml, 3×/day) followed by maintenance during weeks 10–12 (50 ml, 2×/day). Body water enrichment was determined from plasma collected at the time of the OGTT. Tissue and analyte preparation are detailed in the Supporting Information.

### 5.8 Western blotting

The cytoplasmic muscle fraction was used for western blot analysis. 15 µg of protein was loaded on the gel, and proteins were transferred onto PVDF membrane. Kaleidoscope (Bio‐Rad) was used as a molecular weight marker. Blots were incubated overnight with the primary antibodies found in the Supporting Information. Blots were then washed with TBST (3 × 5 min), incubated with anti‐rabbit, HRP‐conjugated secondary antibody, washed with TBST (3 × 5 min) and TBS (2 × 5 min) and detected via chemiluminescence (West Femto, Thermo Scientific). Images were obtained with a UVP bioimaging system, and densitometry was analyzed using Image Studio Lite (version 5.2; Licor).

### 5.9 Statistics

Data are presented as mean ± standard error of the mean (*SEM*), and a significance was set a priori at *p* < 0.05. Normality was confirmed by the D'Agostino and Pearson test. A two‐way ANOVA (treatment group × time) with repeated measures for time was performed. Upon a significant effect, a Holm–Sidak's post hoc test was used for multiple comparisons. When comparing the change (Δ) from pre‐ to postintervention between groups, data were analyzed using an unpaired *t* test. Pearson's correlation coefficient (*r*) was used to determine associations between dependent variables. GraphPad Prism 7.0 was used to create figures and perform statistical analysis.

## CONFLICTS OF INTEREST

The authors have no conflict of interests to disclose.

## AUTHOR CONTRIBUTIONS

ARK, LMB, KLH, and BFM contributed to the design of the work; ARK, JLL, HMS, JJR, WMC, CAW, RVM, and BFM collected the data; ARK, JLL, HMS, WMC, CAW, ODS, MAL, SMB, KLH, and BFM analyzed the data; ARK drafted the manuscript; JLL, JJR, CAW, RVM, ODS, MAL, LMB, SMB, KLH, and BFM critically revised the manuscript; and ARK, JLL, HMS, JJR, WMC, CAW, RVM, ODS, MAL, LMB, SMB, KLH, and BFM approved the agreement to be accountable for all aspects of the work.
