# Supplementary material for: Metformin inhibits mitochondrial adaptations to aerobic exercise training in older adults
Source: Aging Cell. 2018 Dec 11;18(1):e12880. doi: 10.1111/acel.12880 (PMC6351883; doi:10.1111/acel.12880)
Supplement: Supplementary file 1 [file ACEL-18-e12880-s001.docx]

**Supporting Information**

**Participants**

Interested individuals completed a medical history and physical activity questionnaire and a physical exam by the physician medical oversight team. Participants were ineligible if they had mobility limitations, cognitive impairment, or diagnosed with any known cardiovascular, metabolic or kidney disease, including overt T2DM. Additionally, participants were ineligible if they were taking metformin or any other glucose lowering medication, if they were taking medications that may interact with metformin (Dofetilide, Lamotrigine, Pegvisomant, Somatropin, Trimethoprim, Trospium, Gatifloxacin, Cephalexin, Cimetidine, Dalfampridine), if they had received (<6 weeks) or needed imaging that required IV contrast, if they had high blood levels of creatinine (>1.3) or ALT (>52 IU/L), used tobacco or consumed >3 alcoholic beverages per day, had cancer or cancer in remission for <5 years or were on anticoagulant therapy.

**Oral Glucose Tolerance Test**

Blood glucose was analyzed by an automated glucose analyzer (2900 Yellow Springs Instruments, Yellow Springs, CO). Plasma insulin was assessed via a chemiluminescence immune assay (Beckman Coulter). The post OGTT was performed 48 hours after the last exercise bout and 36 hours after the last dose of placebo or metformin. Homeostatic model assessment of insulin resistance was performed using fasting insulin and glucose (HOMA-IR= (Glucose x Insulin)/405; glucose mg/dL; Insulin mU/L). We were unable to place (n=1) or maintain a venous catheter (n=1), nor analyze severely hemolyzed plasma samples (n=1) and therefore no data are available for these OGTT derived measures. One outlier was removed from the Matusda Index in the metformin group because it met two criteria: 1) identified as an outlier via the ROUT method and 2) >2 SD away from the mean.

**Continuous Glucose Monitoring**

A continuous glucose monitor (CGM) (Dexcom, Inc. G6) was placed onto 17 participants by inserting the glucose sensor into the interstitial space of the abdomen adjacent to the umbilical cord. The CGM arm of the study was available for the last 26 individuals, with 17 choosing to participate. The physical and clinical characteristics were not different than the entire study cohort. Participants wore the CGM for 7-10 days before, during week 6 and after the 12-week intervention. While the Dexcom G6 device is FDA approved to monitor glucose without calibrations, we chose to calibrate twice within the first 24 hours and once every 24 hours thereafter by a self-monitored fingerstick blood glucose measurement (Bayer Contour Next). Data was analyzed starting at midnight following the insertion of the sensor and continued for the next 7 days. The publicly available [EasyGV](https://www.phc.ox.ac.uk/research/technology-outputs/easygv) software was used to calculate mean glucose and several indices of glycemic variability.

**Mitochondrial Respiration**

After the biopsy, muscle samples were immediately added to ice-cold BIOPS buffer (2.77 mM CaK_2_-EGTA, 7.23 mM K_2_-EGTA, 20 mM imidazole, 20 mM taurine, 50 mM K-MES, 0.5 mM dithiothreitol, 6.56 mM MgCl_2_, 5.77 mM ATP, and 15 mM phosphocreatine, adjusted to pH 7.1) for mechanical and subsequent chemical permeabilization with saponin (50μg/mL). Blebbistatin, a myosin II-specific inhibitor was added to BIOPS and MiR06 (0.5 mM EGTA, 3 mM MgCl_2_, 60 mM K-lactobionate, 20 mM taurine, 10 mM KH_2_PO_4_, 20 mM HEPES, 110 mM sucrose, 1 g/l BSA essentially fatty acid free, 280 u/ml catalase, pH 7.1) to prevent muscle fiber contraction.

High resolution respirometry was performed on technical duplicates using two different SUIT protocols. The chamber temperature was maintained at 37°C and oxygen was kept between 450 to 300 μM to eliminate oxygen dependence. Both protocols were supported by complex-I linked substrates, pyruvate (5mM), glutamate (10mM) and malate (0.5mM). In SUIT 1, a bolus of ADP (5mM) was added to stimulate maximal complex I-linked OXPHOS (CI_P_). Subsequent additions included cytochrome c (10mM) to test mitochondrial membrane integrity, octanoylcarnitine (0.2mM) to stimulate CI plus fatty acid oxidation supported OXPHOS (CI&FAO_P_), succinate (10mM) for complex I plus II and fatty acid oxidation supported OXPHOS (CI+II&FAO_P_), and FCCP (0.5μM) to stimulate uncoupled electron transport system (ETS; CI+II&FAO_E_) capacity. Next, the complex-I inhibitor rotenone (0.5μM) was added so that the remaining respiration was reflective of CII_E_. Mitochondrial respiration was stopped by the complex III inhibitor antimycin A (2.5μM) to measure residual oxygen consumption (ROX).

For the ADP titration protocol, ADP was injected to reach concentrations of 0.1, 0.175, 0.25, 0.5, 1, 2, 4, 8, 12, and 16 mM followed by sequential addition of cytochrome c, succinate, and FCCP to determine maximal CI+II_P_ and CI+II_E_ as previously performed (Konopka et al. 2017). For both protocols, the ratio of maximal OXPHOS to maximal uncoupled ETS capacity (P/E) was used to gain insight into intrinsic mitochondrial function independent of changes in mitochondrial abundance. Mitochondrial respiration was not available for 3 participants due to technical difficulties.

**Telomere Length Analysis**

Multiplexed qPCR measurements of telomere length were carried out as previously described (Cawthon 2009). Here, a 22 μL master mix was prepared using SYBR green GoTaq qPCR master mix (Promega #A6001) combined with the telomere and albumin forward primer and telomere reverse primers (shown in supporting information) at 10 μM per primer (Integrated DNA Technologies), and RNase/DNase free water. To the master mix, 3 μL of DNA at 3.33 ng/uL was added for a final volume of 25 μL. The TelG/C primers were at a final concentration of 900 nM and the AlbU/D primers at 400 nM.

Primers for forward (TelG; 5’ACACTAAGGTTTGGGTTTGGGTTTGGGTTTGGGTTAGTGT-3’) and reverse telomere (TelC; 5’-TGTTAGGTATCCCTATCCCTATCCCTATCCCTATCCCT AACA-3’) and forward (AlbU; 5’-CGGCGGCGGGCGGCGCGGGCTGGGCGGA AATGCTGCACAGAATCCTTG-3’) and reverse albumin (AlbD; 5’-GCCCGGCCCGCCG CGCCCGTCCCGCCGGAAAAGCATGGTCGCCTGTT-3’).

The cycle parameters were set as follows: 95°C for 3 min; 94°C for 15 s, 49°C for 15 s, for 2 cycles; 94°C for 15 s, 62°C for 10 s, 74°C for 15 s, 84°C for 10 s, and 88°C for 15 s, for 32 cycles. The melting curve was established by a 72°C to 95°C ramp at 0.5°C/second increase with a 30 second hold.

**Tissue and Analyte Preparation for Gas Chromatography Mass Spectrometry (GCMS)**

Body water enrichment was determined from plasma as we have previously performed (Robinson et al. 2011; Miller et al. 2012). Using differential centrifugation, skeletal muscle was fractionated to measure protein synthesis rates of subcellular fractions enriched with myofibrillar, cytoplasmic, and mitochondrial proteins according to our previously published standard operating procedures (Robinson et al. 2011; Miller et al. 2012). Following tissue fractioning, analytes were prepared for analysis on a 7890A gas chromatograph coupled to a 5975C mass spectrometer with a DB-5MS GC column (30 m × 0.25 mm × 0.25 μm; all from Agilent).

**Western Blotting Antibodies**

Western blotting was performed in a subset of participants due to limited tissue availability (PLA: n=15; MET: n=18). All antibodies are from Cell Signaling Technology: pAMPK (1:800 primary, 1:1000 secondary; #2531), AMPK (1:500 primary, 1:2500 secondary; #2532), p4EBP (1:750 primary, 1:2500 secondary; #9459), 4EBP (1:500 primary, 1:2500 secondary; #9452). pRPS6 (1:1000 primary, 1:5000 secondary; #2217), RPS6 (1:500 primary, 1:1000 secondary; #4858), pAKT T308 (1:500 primary, 1:1000 secondary; #2965), pAKT s473 (1:500 primary, 1:1000 secondary; #4058), AKT (1:500 primary, 1:1000 secondary; #4865).

**Supporting Information References**

Cawthon RM (2009) Telomere length measurement by a novel monochrome multiplex quantitative PCR method. *Nucleic Acids Res.* 37, e21.

Konopka AR, Castor WM, Wolff CA, Musci RV, Reid JJ, Laurin JL, Valenti ZJ, Hamilton KL & Miller BF (2017) Skeletal muscle mitochondrial protein synthesis and respiration in response to the energetic stress of an ultra-endurance race. *J. Appl. Physiol. Bethesda Md 1985* 123, 1516–1524.

Miller BF, Robinson MM, Bruss MD, Hellerstein M & Hamilton KL (2012) A comprehensive assessment of mitochondrial protein synthesis and cellular proliferation with age and caloric restriction. *Aging Cell* 11, 150–161.

Robinson MM, Turner SM, Hellerstein MK, Hamilton KL & Miller BF (2011) Long-term synthesis rates of skeletal muscle DNA and protein are higher during aerobic training in older humans than in sedentary young subjects but are not altered by protein supplementation. *FASEB J.* 25, 3240–3249.

| Supplemental Table 1 | Carbohydrate | | | | Protein | | | |  |  |  | |
| --- | --- | --- | --- | --- | --- | --- | --- | --- | --- | --- | --- | --- |
|  | **PLA** | | **MET** | | **PLA** | | **MET** | | **P Values** | | | |
|  | PRE | POST | PRE | POST | PRE | POST | PRE | POST | Time | Treatment | | Interaction |
| N | 12 (10 W, 2 M) | | 14 (11 W, 3 M) | | 14 (11 W, 3 M) | | 13 (10 W, 3 M) | |  |  | |  |
| Age (years) | 64±2 | | 61±1 | | 62±1 | | 62±1 | |  |  | |  |
| Body Weight (kg) | 87±4 | 85±4 | 85±5 | 83±5 | 82±6 | 82±6 | 86±5 | 84±6 | **P=0.009** | P=0.97 | | **P=0.04** |
| BMI (kg/m^2^) | 31±2 | 29±2 | 30±2 | 29±2 | 30±2 | 30±2 | 32±2 | 31±2 | **P<0.001** | P=95 | | P=0.21 |
| Fat (kg) | 35±3 | 34±2 | 34±3 | 32±3 | 33±4 | 32±4 | 33±3 | 31±3 | **P<0.001** | P=0.97 | | P=0.36 |
| FFM (kg) | 48±3 | 48±2 | 48±3 | 47±2 | 46±2 | 46±2 | 50±2 | 50±3 | P=0.97 | P=0.80 | | P=0.08 |
| Trunk Fat (kg) | 17±2 | 16±1 | 17±2 | 16±2 | 17±3 | 16±2 | 18±2 | 17±2 | **P<0.001** | P=0.96 | | P=0.95 |
| Leg Fat (kg) | 12±1 | 12±1 | 12±1 | 11±1 | 11±2 | 11±2 | 11±1 | 10±1 | **P=0.01** | P=0.84 | | P=0.81 |
| Leg FFM (kg) | 15±1 | 15±1 | 15±1 | 14±1 | 15±1 | 15±1 | 16±1 | 16±1 | P=0.93 | P=0.69 | | P=0.46 |
| RER | 1.21±0.02 | 1.18±0.02 | 1.22±0.02 | 1.15±0.01 | 1.16±0.02 | 1.15±0.02 | 1.17±0.02 | 1.15±0.02 | **P=0.004** | P=0.23 | | P=0.12 |
| HbA1c (%) | 5.7±0.1 | 5.6±0.1 | 5.6±0.1 | 5.5±0.1 | 5.8±0.1 | 5.7±0.1 | 5.8±0.1 | 5.7±0.1 | **P=0.002** | P=0.17 | | P=0.65 |
| Fasting Glucose (mg/dL) | 97±2 | 95±2 | 101±4 | 100±3 | 96±2 | 95±2 | 108±4 | 105±3 | P=0.26 | **P=0.01** | | P=0.90 |
| Fasting Insulin (𝜇IU/mL) | 8.3±1.8 | 5.8±0.6 | 12.5±3.8 | 10.6±3.2 | 8.1±1.5 | 6.4±1.2 | 7.8±1.6 | 5.7±1.6 | **P=0.01** | P=0.30 | | P=0.95 |
| HOMA-IR | 2.0±0.4 | 1.2±0.1 | 3.0±1.1 | 2.4±0.8 | 1.6±0.4 | 1.3±0.3 | 1.8.0±0.4 | 1.2±0.3 | **P=0.01** | P=0.27 | | P=0.91 |
| Glucose AUC | 18148±742 | 16843±548 | 19178±972 | 19929±728 | 16021±661 | 16159±867 | 18788±1130 | 19363±883 | P=0.91 | **P=0.005** | | P=0.25 |
| Insulin AUC | 7501±1866 | 7964±2856 | 9397±3009 | 9729±4709 | 8567±2951 | 7012±2305 | 5695±1453 | 5988±1157 | P=0.66 | P=0.20 | | P=0.31 |
| 2-hr Glucose (mg/dL) | 116±7 | 115±7 | 131±7 | 133±7 | 102±7 | 107±10 | 108±14 | 112±12 | P=0.51 | P=0.17 | | P=0.94 |
| 2-hr Insulin (𝞵IU/mL) | 57±14 | 51±8 | 86±20 | 91±34 | 56±13 | 55±15 | 39±7 | 32±7 | P=0.64 | P=0.16 | | P=0.89 |

FFM, fat free mass; HR, heart rate; RER, Respiratory Exchange Ratio at VO_2max_, HOMA-IR, homeostatic model of insulin resistance; AUC, area under the curve
